# Professional dental prophylaxis increases salivary cortisol in children with dental behavioural management problems: a longitudinal study

**DOI:** 10.1186/s12903-016-0273-1

**Published:** 2016-08-18

**Authors:** Heloisa Sousa Gomes, Liliani Aires Candido Vieira, Paulo Sucasas Costa, Aline Carvalho Batista, Luciane Rezende Costa

**Affiliations:** 1 Programa de Pós-Graduação em Odontologia, Universidade Federal de Goiás (UFG), Av. Universitária Esquina com 1ª Avenida s/n, Setor Universitário, CEP: 74605-220 Goiânia, GO Brazil; 2 Departamento de Saúde Oral, Faculdade de Odontologia/UFG, Av. Universitária Esquina com 1ª Avenida s/n, Setor Universitário, CEP: 74605-220 Goiânia, GO Brazil; 3 Departamento de Pediatria, Faculdade de Medicina/UFG, Rua 235 c/ 1a. s/n - S. Universitário, CEP 74605-020 Goiânia, GO Brazil; 4 Departamento de Ciências Estomatológicas, Faculdade de Odontologia/UFG, Av. Universitária Esquina com 1ª Avenida s/n, Setor Universitário, CEP: 74605-220 Goiânia, GO Brazil; 5 Faculdade de Odontologia, Universidade Federal de Goiás, Primeira Avenida, Setor Universitário, CEP: 74605-220 Goiânia, Goiás Brazil

**Keywords:** Dental care for children, Stress, psychological, Saliva, Dental prophylaxis, Child behaviour

## Abstract

**Background:**

Dental procedures may cause stress and increase the salivary cortisol levels. It is important to known if apparently simple procedures such as professional dental prophylaxis at low speed (DP) are stressful for children with dental behaviour management problems (DBMP) to help with behaviour guidance strategies. This longitudinal study aimed to evaluate if DP changes a physiological marker of stress (salivary cortisol) in children with DBMP who were referred to dental treatment under sedation.

**Methods:**

One paediatric dentist carried out a DP with rubber cup and pumice followed by dental examination in 39 children aged 2–5 years, prior to the dental sedation appointment. Children’s saliva was collected at three different moments: upon waking (UW), on arrival at the dental office reception area (RA) and 25 min after the dental prophylaxis (DP). The saliva samples were analysed using an enzyme immunoassay kit. The Wilcoxon test was used in paired comparison (*P* < 0.05).

**Results:**

Salivary cortisol levels decreased from UW (0.34; 0.15–0.54) to RA (0.14; 0.08–0.56) (*P* = 0.019) and increased from RA to DP (0.25; 0.06–1.48) (*P* = 0.008). Higher salivary cortisol levels were observed at DP when compared to RA in children who did not have previous dental treatment (*P* = 0.007), had toothache (*P* = 0.006), presented some protest behaviour during DP (*P* = 0.008), or needed protective stabilisation by parents for the dental examination (*P* = 0.005).

**Conclusions:**

Paediatric dentists should be aware that even simple procedures such as professional dental prophylaxis are related to stress in young children.

## Background

Dental fear/anxiety and dental behaviour management problems (DBMP) affect 9 % of the child and adolescent population [1]. Children with DBMP are referred to specialists in paediatric dentistry [2] mainly for reasons of temperament [3] and experience greater dental fear levels than children in ordinary dental care [4] even during usual dental procedures [5]. Dental caries is associated with dental anxiety [6] probably because children with toothache are more anxious [7–9] than those who have never experienced dental pain [8]. In another hand, dental anxiety is associated with children who have never experienced a dental appointment [7].

Different scales have been used to evaluate children’s psychological characteristics such as behaviour and anxiety during dental procedures [10], but these measures are subjective. Salivary biomarkers have great physiological research interest for accessing stress-producing events [11]. Stressor stimulus activates the hypothalamic-pituitary-adrenal axis (HPA) [12] which results in an increased secretion of cortisol, a biomarker, into serum, urine [13] and saliva [14–16]. In fact, cortisol in the saliva of children undergoing dental treatment is a physiological measure associated with stress [14, 16, 17] and dental anxiety [9, 18]. Thus, assessing stress through salivary cortisol may be preferable to serum or urine because it is an easy, safe, non-invasive and painless method [19].

The clinical reason for measuring salivary cortisol in children in the dental setting is as follows: cortisol is related to dental stress and anxiety [9, 14, 16–18]; dental anxiety is associated with pain [7, 8] and DBMP [1, 20]; thus, salivary cortisol could be an objective tool for the diagnosis of DBMP and pain complementing subjective evaluations such as self-report and observational scales. By detecting that a child has a high salivary cortisol, we can better manage the dental appointment in order to provide the most comfortable experience possible.

According to the literature results on salivary cortisol in children with or without dental caries, dental treatment can be stressful in a first dental appointment [14, 15], during restorative treatment [17, 18], or even in anticipation of an event during the dental session [16, 18]. Dental prophylaxis (DP) removes plaque [21], facilitates clinical examinations and introduces dental procedures to the child [22]. However, there is a lack of information on how stressful DP can be in children with history of DBMP. The aim of this study was to assess salivary cortisol levels and associated factors in young children with history of DBMP undergoing professional dental prophylaxis for dental examination. The hypothesis was that DP changes the salivary cortisol levels of children with DBMP.

## Methods

### Study ethics, design and setting

This longitudinal study, approved by the Institutional Research Ethics Board of the Federal University of Goias, Brazil (protocol #307/2011), was carried out in a university dental sedation centre, which has the mission of providing dental treatment under sedation for referred people in an outpatient basis. The World Medical Association Declaration of Helsinki principles and national requirements were followed. The children’s parents signed the consent form after a through explanation about the study. The recruitment and data collection were done between April 2012 and December 2012.

### Participants

Participants were 2 to 5-year-olds children referred for dental treatment under sedation due to DBMP in previous dental appointments in public primary care services. Sample size was calculated on www.statstodo.com using the requirements for paired difference studies. Once the hypothesis was that professional dental prophylaxis changes children’s cortisol levels, the variables considered were the salivary cortisol at the moments “arrival at the dental office reception area” (RA) and “25 min after dental prophylaxis” (DP). The provided parameters for calculation were: significance level (adjusted for sidedness) = 0.025, power = 0.8, standard deviation of the difference = 0.4 and difference in means = 0.2. The last two values are meaningful and were obtained by compilation of data from reference #15, which has similarities with the present study. So, a sample of 34 patients was estimated. Considering a possible loss of saliva samples, this study was carried out with a sample of 39 children referred for dental treatment under sedation and included by nonprobability sampling. All the children had attended another dentist before the dental appointment but 32 (82.1 %) had not received any dental treatment because of their negative behaviour.

### Clinical procedures

One specialist in paediatric dentistry carried out a standard dental examination in 39 children during a morning appointment. It began with prophylaxis with pumice in a rubber cup at low speed. None of the children were sedated during the prophylaxis or dental examination. An observer recorded the dental caries activity using the decayed, missing, or filled primary teeth (dmft) index of the World Health Organization (WHO) [23]. Children who did not cooperate were stabilised by a parent, who was present for the entire procedure and sat in the dental chair with the child.

Information reported by parents about children who had previous dental treatment and toothache during the last month was recorded in the specific form by the observer at the day of the dental examination. At the end of the procedure a specialist in paediatric dentistry evaluated the clinical behaviour during the dental examination according to the Brazilian version of the Venham Behavior Rating Scale [10] (0 – total cooperation; 1 – moderate protest; 2 – intense protest; 3 – more intense protest; 4 – generalised protest) [10].

Three samples of saliva were collected using Salivette tubes (Sarstedt Inc., Nümbrecht, Germany). The principal researcher collected children’s saliva at their home, upon waking (UW) in a leisurely day other than the day of the dental appointment to avoid any influence on cortisol awakening response. The researcher took additional saliva samples at two other moments on the day of the procedure: one immediately on arrival at the dental office reception area (RA) and the other, 25 min after dental prophylaxis (DP). This 25 min lag is required to cortisol reach its peak in saliva after contact with the stressor [24].

### Laboratory procedures

After saliva collection, the Salivette® tubes were centrifuged at 3000 rpm for 15 min (Sislab/Basic, São Paulo, SP, Brazil). They were subsequently stored in Eppendorf tubes and frozen at −80° (Sanyo/Vip® Plus™, Wood Dale, Illinois, USA) until the time of analysis. The cortisol in the saliva samples was measured using an enzyme immunoassay kit (Salimetrics, State College, PA, USA). Samples were evaluated in duplicate using a microplate reader (Molecular Devices, Spectra Max 190, Sunnyvale, CA, USA) for absorbance at 450 nm. Cortisol levels were determined in accordance with the standard curves prepared following the manufacturer’s instructions. The detection limits varied from 0.012 μg/dL to 3000 μg/dL.

### Statistical methods

Data were entered and analysed using the IBM SPSS 22.0 (IBM Corporation, New York, NY, USA) and Prism software (GraphPad Prism 5; GraphPad Software, San Diego, CA, USA). The Shapiro-Wilk test was used to analyse the normality of data.

Salivary cortisol levels in the three established moments (UW, RA and DP) were compared. Paired comparison was made between RA and DP regarding children’s dental history aspects (previous dental treatment attempt, toothache) and current status (dental treatment needs, protest behaviour and necessity of protective stabilisation by parents during consultation). The Wilcoxon test was used in both analyses and a *P*-value less than 0.05 was considered as statistically significant.

## Results

A total of 39 children were included in this study (53.8 % girls), with a mean age of 45.1 (SD 14.3) months and presenting mean dmft of 7.15 (SD 4.58). The duration of dental examination was 10.4 (SD 3.6) minutes. Of the 39 children evaluated, 36 (92.3 %) presented with tooth decay needing restoration, pulp therapy or extraction. According to the Brazilian version of the Venham Behavior Rating Scale [10], 26 (66.6 %) children presented some protest behaviour (scores 1, 2, 3 or 4) during the dental examination. Lest than half of the children (43.6 %) needed to be restrained by parents to have the dental examination concluded.

**Fig. 1:**
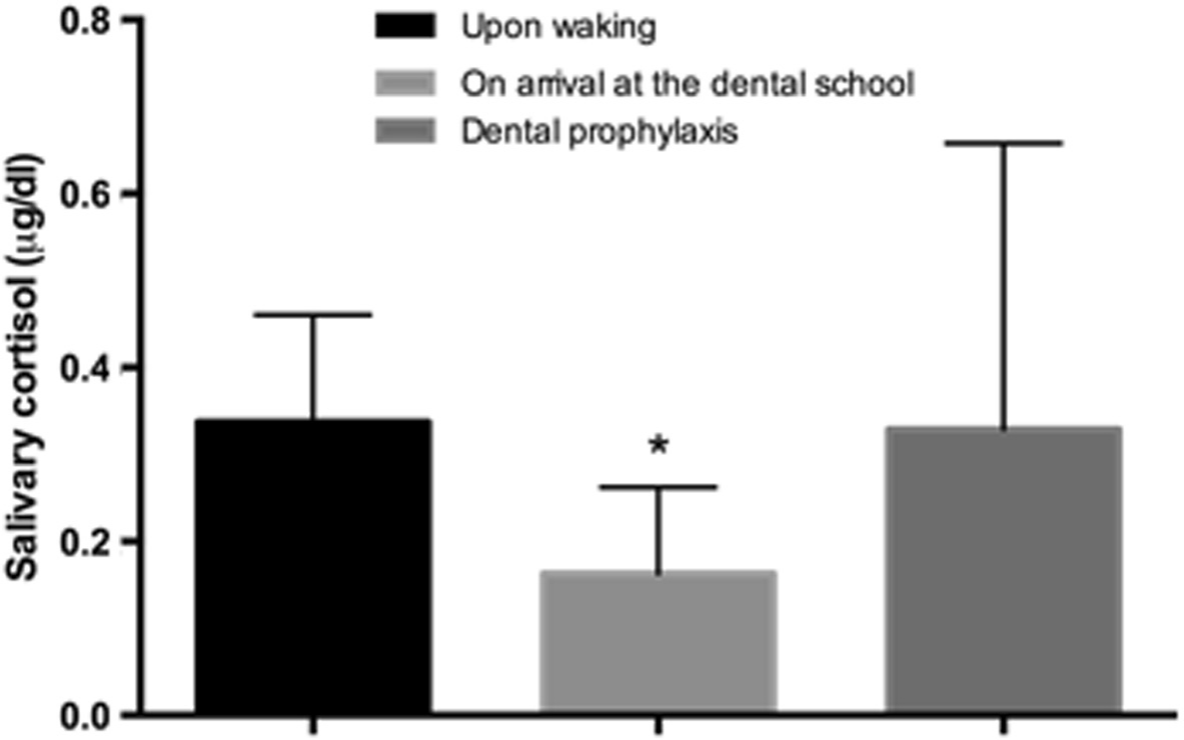
Level of salivary cortisol in children, paired comparison between different moments of collection, upon waking (UW), on arrival at the dental office reception area (RA) and at the time of dental prophylaxis (DP). * statistical difference between RA and UW (*P* = 0.019); RA and DP (*P* = 0.008)

Salivary cortisol levels varied throughout the dental examination and did present a non normal distribution (Shapiro-Wilk test, *P* < 0.05). A paired comparison of salivary cortisol levels in the children showed that at the moment of waking [median (minimum-maximum)] [0.34 μg/dl (0.15–0.54)] and at the moment of dental prophylaxis [0.25 μg/dl (0.06–1.48)], the cortisol levels were higher than at the moment of arrival at the dental office [0.14 (0.081–0.561)]. These differences were significant at both moments (Wilcoxon, *P* = 0.019 and *P* = 0.008, respectively) (Fig. 1).

Salivary cortisol levels were higher at DP than at RA in children: who did not receive any previous dental treatment (*P* = 0.007), whose parents reported children had toothache (*P* = 0.006), who had some protest behaviour (*P* = 0.008) and who needed protective stabilisation (*P* = 0.005) (Table 1).

**Table 1:** Salivary cortisol levels in children during dental examination

Children	Number	Salivary cortisol (μg/dL)		*P* value*
		Median (minimum-maximum)		
		Dental office reception area	Dental prophylaxis	
Gender				
Female	21	0.11 (0.06–0.33)	0.22 (0.10–0.61)	0.27
Male	18	0.15 (0.09–0.56)	0.25 (0.02–1.48)	0.92
Age				
2–3 years	23	0.14 (0.06–0.56)	0.24 (0.02–1.48)	0.62
4–5 years	16	0.12 (0.07–0.33)	0.22 (0.10–0.61)	0.84
Dental caries activity				
Without pulp exposure	18	0.23 (0.09–0.56)	0.26 (0.23–0.29)	0.14
With pulp exposure	22	0.14 (0.08–0.32)	0.22 (0.06–1.48)	0.04**
Previous dental treatment				
Yes	7	0.11 (0.11–0.16)	0.15 (0.06–1.48)	0.89
No	32	0.14 (0.06–0.56)	0.24 (0.02–1.28)	0.007**
Toothache				
Yes	28	0.14 (0.06–0.33)	0.24 (0.02–1.48)	0.006**
No	11	0.11 (0.09–0.56)	0.21 (0.08–0.76)	0.57
Protest (scores 1, 2, 3 or 4)				
Yes	26	0.13 (0.06–0.32)	0.26 (0.02–1.48)	0.008**
No	13	0.12 (0.07–0.56)	0.18 (0.09–0.3)	0.38
Protective stabilisation by parents				
Yes	17	0.11 (0.08–0.23)	0.46 (0.02–1.48)	0.005**
No	22	0.14 (0.06–0.56)	0.16 (0.06–0.35)	0.54

*Wilcoxon test

**Represents a statistically significant difference in salivary cortisol levels

## Discussion

Dental prophylaxis is considered an introductory non-stressful procedure for children with no history of dental treatment experience [14, 16] and no dental pain [16]. Conversely, this study shows that prophylaxis with a rubber cup at low speed increases salivary cortisol levels, a stress biomarker, in young children with DBMP. Also, children’s stress is associated with toothache and no previous dental treatment, as well as with protest behaviour and protective stabilisation by parents during the dental examination.

The circadian rhythm shows that a person’s highest level of cortisol occurs upon waking and then decreases throughout the day [25] until evening. Thus, the cortisol level at the most stressful moment of dental treatment is compared with that of waking up [16] as shown in other studies [16, 26]. According to our findings, the DP level of cortisol was similarly compared to that of UW, but on the contrary, Furlan et al. showed that the highest level of salivary cortisol was before the dental examination and this was also compared with waking up time [16]. However, their research involved cooperative children under seven, who did not have toothache or cavities and who were going to the dentist for the first time [16].

Yfanti et al. found higher salivary cortisol levels after dental procedures such as dental prophylaxis or restorations with local anaesthesia in children aged 6–10 years who had already received some earlier dental treatment [18]. In fact, local anaesthesia injection can rise the salivary cortisol level in children [17]. On the other hand, other studies have found higher cortisol levels before the dental prophylaxis, but their participants were cooperative children [16] or children who had never visited a dentist [14–16].

This study found that salivary cortisol increased during dental prophylaxis in children who did not allow dental treatment to be performed on a previous occasion because of DBMP. This was expected, because children who had already received some dental treatment would be more able to cope with stressful stimuli throughout the treatment sessions [6, 8, 15]. Blomqvist et al. reported no change in the cortisol levels of 13-year-olds during a dental visit [26]. However, in their study patients underwent clinical and radiographic exam without any other dental procedure [26]. Besides, our findings show an association between increased cortisol and children who had some behavioural problems during the dental examination. Dental anxiety is also associated with higher levels of salivary cortisol in children who had already undergone dental treatment [15–18] and children who had shown negative behaviour during treatment [27].

Our results showed high salivary cortisol levels in children who had caries without pulp exposure in both RA and DP, whereas dental prophylaxis was related to higher stress in children who presented caries with pulp exposure. There association between cortisol and dental caries is debated [15, 28], but probably the change in cortisol level observed here is mainly related to the stress of the procedure [15, 17] and probably by the presence of pain [7–9] in children with pulp exposure. We also observed that children with toothache had increased cortisol levels at DP.

Because of the challenging behaviour of most children during dental prophylaxis in our study, many accompanying parents had to protectively stabilise their children to have the dental examination performed. Thus, this study showed that children, referred for dental treatment under sedation because of behaviour management problems, experience stress during dental prophylaxis with a rotating device. This stress can be expressed in behavioural protest, such as crying or movement, which disrupts treatment and obliges parents to protectively stabilise their children for the dental examination be properly and safely completed.

The children in this study had an earlier consultation with a dentist but not all received dental treatment due to negative behaviour. So, one major limitation of this study is that some subgroups had a small sample (e.g. children who had successful dental treatment before), and a type II error might be occurred. Although this study elucidated the dental prophylaxis as a stressor compared to earlier moments, having a child as his/her own control, another possible limitation was the lack of comparison to a group of children without DBMP. Thus, further studies are needed to investigate cortisol in larger groups of children as well as comparing to children who cooperate in the dental setting. Besides, another study could compare professional dental prophylaxis versus toothbrush cleaning for the dental examination in causing stress in children.

All in all, our findings show that dental prophylaxis with a rubber cup at low speed triggers stress in children, as assessed by the level of salivary cortisol, a stress biomarker. Toothbrush prophylaxis and rubber cup prophylaxis have the same goals, namely to remove plaque, make patients acquainted with the dental environment and facilitate examination. The difference between those two methods is that the rubber cup can remove stains from the patient’s teeth. The prophylaxis with a rotating device for this group of children should be reviewed; maybe teeth cleaning with a toothbrush would be less harmful.

## Conclusions

Professional dental prophylaxis with pumice in a rubber cup at low speed was related to stress in children with history of dental behaviour management problems. Also, salivary cortisol levels (i.e. stress) during professional dental prophylaxis increased in children: who had dental caries activity with pulp exposure; who did not successfully complete a previous dental treatment because of dental behaviour management problem; with toothache report; who present protest behaviour and/or need to be protectively stabilised by parents during a dental clinical examination.

## Abbreviations

DBMP: Dental behaviour management problems
DMFT: Decayed, missing, or filled primary teeth
DP: Dental prophylaxis
HPA: Hypothalamic-pituitary-adrenal
RA: Arrival at the dental office reception area
SD: Standard deviation
UW: Upon waking
WHO: World Health Organization
